# Circulating cell-free miR-494 and miR-21 are disease response biomarkers associated with interim-positron emission tomography response in patients with diffuse large B-cell lymphoma

**DOI:** 10.18632/oncotarget.26141

**Published:** 2018-10-05

**Authors:** Qingyan Cui, Frank Vari, Alexandre S. Cristino, Carlos Salomon, Gregory E. Rice, Muhammed B. Sabdia, Dominic Guanzon, Carlos Palma, Marina Mathew, Dipti Talaulikar, Sanjiv Jain, Erica Han, Mark S. Hertzberg, Clare Gould, Pauline Crooks, Gayathri Thillaiyampalam, Colm Keane, Maher K. Gandhi

**Affiliations:** ^1^ University of Queensland Diamantina Institute, Brisbane, QLD, Australia; ^2^ University of Queensland Centre for Clinical Research, Brisbane, QLD, Australia; ^3^ University of Concepción, Concepción, Chile; ^4^ Canberra Hospital, Garran, ACT, Australia; ^5^ Australia National University Medical School, Garran, ACT, Australia; ^6^ Prince of Wales Hospital, Sydney, NSW, Australia; ^7^ Princess Alexandra Hospital, Brisbane, QLD, Australia

**Keywords:** miRNA-494, miRNA-21, positron emission tomography, diffuse large B-cell lymphoma, biomarker

## Abstract

MicroRNA (miRNA)s are dysregulated in Diffuse large B-cell lymphoma (DLBCL), where they reflect the malignant B-cells and the immune infiltrate within the tumor microenvironment. There remains a paucity of data in DLBCL regarding cell-free (c-f) miRNA as disease response biomarkers. Immunosuppressive monocyte/macrophages, which are enriched in DLBCL, are disease response markers in DLBCL, with miRNA key regulators of their immunosuppressive function. Our aim was to determine whether plasma miRNA that reflect the activity of the malignant B-cell and/or immunosuppressive monocytes/macrophages, have value as minimally-invasive disease response biomarkers in DLBCL.

Quantification of 99 DLBCL tissues, to select miRNA implicated in immunosuppressive monocytes/macrophage biology, found miR-494 differentially elevated. In a discovery cohort (22 patients), pre-therapy c-f miR-494 and miR-21 but not miR-155 were raised relative to healthy plasma. Both miR-494 and miR-21 levels 3-6 months reduced post immuno-chemotherapy. The validation cohort (56 patients) was from a prospective clinical trial. Interestingly, in sequential samples both miRNAs decreased in patients becoming Positron Emission Tomography/Computerized Tomography (PET/CT)-ve, but *not* in those remaining interim-PET/CT+. Patient monocytes were phenotypically and functionally immunosuppressive with *ex-vivo* monocyte depletion enhancing T-cell proliferation in patient but not healthy samples. Pre-therapy monocytes showed an immunosuppressive transcriptome and raised levels of miR-494. MiR-494 was present in all c-f nanoparticle fractions but was most readily detectable in unfractionated plasma.

Circulating c-f miR-494 and miR-21 are disease response biomarkers with differential response stratified by interim-PET/CT in patients with DLBCL. Further studies are required to explore their manipulation as potential therapeutic targets.

## INTRODUCTION

Diffuse large B-cell lymphoma (DLBCL) is the most common Non-Hodgkin's Lymphoma (NHL), accounting for ~40% of new cases [1, 2]. It is a heterogeneous disease with highly variable molecular features present in both the malignant B-cell [3–5], and the tumor microenvironment (TME) [6, 7]. Further understanding of DLBCL pathobiology is required for precision medicine utilizing selective use of highly-targeted therapies to be best applied.

MicroRNAs (miRNAs) are ~21 to 23-nucleotide-long noncoding RNA molecules that bind to specific cognate sequences in the 3′-untranslated regions (3′-UTR) of target transcripts [8]. The resultant translational repression and gene silencing that ensues enables them to play key roles in numerous biologic processes by fine tuning the expression of a variety of protein-coding genes. MiRNAs are ubiquitously dysregulated in a range of diseases and malignancies including DLBCL [9–11], and are enriched in a variety of cell types that are over-represented within the TME [12]. Not only are miRNA believed to both reflect and contribute to the underlying pathobiology of the aggressive B-cell lymphoma sub-type Diffuse Large B-cell Lymphoma (DLBCL), but they hold promise as both potential therapeutic targets and minimally-invasive circulating biomarkers [13, 14].

Cell-free (c-f) miRNA are remarkably stable in plasma and/or serum and are resistant to multiple freeze–thaw cycles, and a variety of groups have investigated the role of c-f miRNA as circulating biomarkers that reflect disease activity [15]. We have previously shown that several plasma miRNA, including miR-494, miR-155 and miR-21, are elevated in classical Hodgkin Lymphoma (cHL) [16], a histopathological atypical neoplastic B-cell entity that contains only ~2% of malignant (Hodgkin Reed-Sternberg ‘HRS’) cells within the diseased node. Interestingly, miR-155 and miR-21 *but not* miR-494 have been shown to be highly expressed in micro-dissected HRS cells [17]. One explanation for this finding, is that non-HRS cells present within the TME are also contributing as a major source of plasma miRNA. Consistent with this, in cHL - a setting in which circulating monocytoid myeloid-derived-suppressor cells ‘moMDSCs’ and tumor associated macrophages ‘TAMs’ are elevated [18], we have shown that the concentrations of soluble CD163 (a circulating marker of immunosuppressive monocytes) are differentially reduced in those entering interim radiographic complete remission (CR) versus those with only partial response to chemotherapy [19].

Microarray analysis of DLBCL cell-lines, reports that the expression of miR-155 and miR-21 is also raised compared with normal peripheral blood B-cells [20]. There is no data for miR-494 however it is known to be differentially over-expressed in the tissue of the most common indolent NHL subtype follicular lymphoma [21]. Plasma miR-155 and miR-21 are also known to be elevated at diagnosis in DLBCL. However, there is minimal data regarding the expression of these miRNA in the circulation during and after therapy, or of their kinetics in comparison to interim Positron Emission Tomography/Computerized Tomography (interim-PET/CT) scans.

It has been consistently shown that moMDSCs contribute to the negative regulation of T-cell effector immunity in a variety of malignancies [22]. In patients with DLBCL, we and others have shown that the circulating CD14+HLA-DR^lo^ moMDSC subset is associated with higher rates of disease progression and distinct kinetics after treatment with immuno-chemotherapy [23–25]. Soluble CD163 is also elevated at diagnosis in DLBCL and declines in response to therapy [23]. Notably the expression of specific miRNA within immunosuppressive monocytes/macrophages, including miR-494, miR-155 and miR-21 are induced by tumor-derived factors, leading to the accumulation and functional enhancement of tumor-expanded MDSCs in model systems [26, 27]. Collectively, these results suggest that plasma miRNA that reflect the activity of the malignant B-cell and/or immunosuppressive monocytes/macrophages may have value as minimally-invasive biomarkers in DLBCL.

The intention of this study was to address the role of plasma miRNA as circulating biomarkers that are enriched in malignant B-cells and/or moMDSC miRNA in patients treated with conventional first-line ‘R-CHOP’ (rituximab, cyclophosphamide, doxorubicin, vincristine, prednisolone) immuno-chemotherapy in DLBCL. A conventional discovery/validation patient cohort approach was utilized, in which the validation cohort were drawn from a PET/CT risk-adapted prospective clinical trial. A variety of techniques were used including digital multiplex and real-time PCR based gene expression in tissues and plasma, nanoparticle testing and functional immunoassays.

## RESULTS

### Selection of candidate moMDSC-related miRNA by tissue miRNA array

Initial experiments were aimed to identify candidate miRNA in tissue samples that could then be further evaluated as an exemplar of a moMDSC-related miRNA circulating biomarker. Tissue quantification used two non-overlapping methodologies applied to diagnostic FFPE tissue DLBCL biopsies. Initially, RNA was quantified using the Agilent miRNA microarray platform in the tissue samples of 23 patients with DLBCL and 8 lymph nodes from adults without lymphoma (tissue discovery cohort). DLBCL tissues regions for extraction were pre-selected by a hematopathologist to ensure that only areas of lymphoma involvement were extracted, but no microdissection (i.e. the tissue was a mixed infiltrate) for either malignant or non-malignant cell enrichment was applied. Eight miRNAs previously implicated in moMDSC biology, miR-223, miR-200c, miR-17-5p, miR-20a, miR-494, miR-21, miR-155 and miR-101 [26, 27], were interrogated for differential-expression (DE). This showed that only three miRNAs were identified as being DE: miR-494 and miR-200c (over), and miR-223 (under). For tissue validation we used 76 DLBCL and 12 non-lymphomatous nodes by using nanoString nCounter platform. There was no overlap between the discovery and validation tissue cohorts. Only miR-494 was consistently (over) DE by both miRNA quantification platforms (Table 1). MiR-494 was chosen for further evaluation as the archetypal exemplar of moMDSC-related miRNA. Despite not showing consistent DE, miR-21 and miR-155 were also included due to the findings in previous studies that showed they are enriched in DLBCL cell-lines and are elevated in the circulation at diagnosis [13, 28–33].

**Table 1:** Differential expression of moMDSC associated miRNA in discovery and validation DLBCL tissue cohorts

moMDSC associated miRNA	Agilent array		NanoString array	
	Fold change	Adj. *P* value	Fold change	Adj. *P* value
**miR-494**	23.10	0.005	6.20	<0.001
**miR-223**	0.47	0.016	1.36	NS
**miR-200c**	0.12	0.026	19.52	NS
**^1^miR-17-5p**	1.68	NS	2.55	0.005
**^2^miR-20a**	1.32	NS	1.96	NS
**miR-21**	1.27	NS	1.31	NS
**miR-155**	2.75	NS	5.04	<0.001
**miR-101**	0.59	NS	ND	NA

*P* values were adjusted for Benjamini-Hocheberg multiple hypothesis testing. NS, not significant. NA, not applicable. ^1^The nanoString probe for miR-17-5p was unable to distinguish between miR-17-5p and miR-106a-5p. ^2^ The nanoString probe for miR-20a was unable to distinguish between miR-20a-5p and miR-20b-5p.

### Plasma miR-494 and miR-21 are disease response biomarkers in patients with DLBCL and the kinetics of their decline is associated with interim-PET/CT response

**Figure 1:**
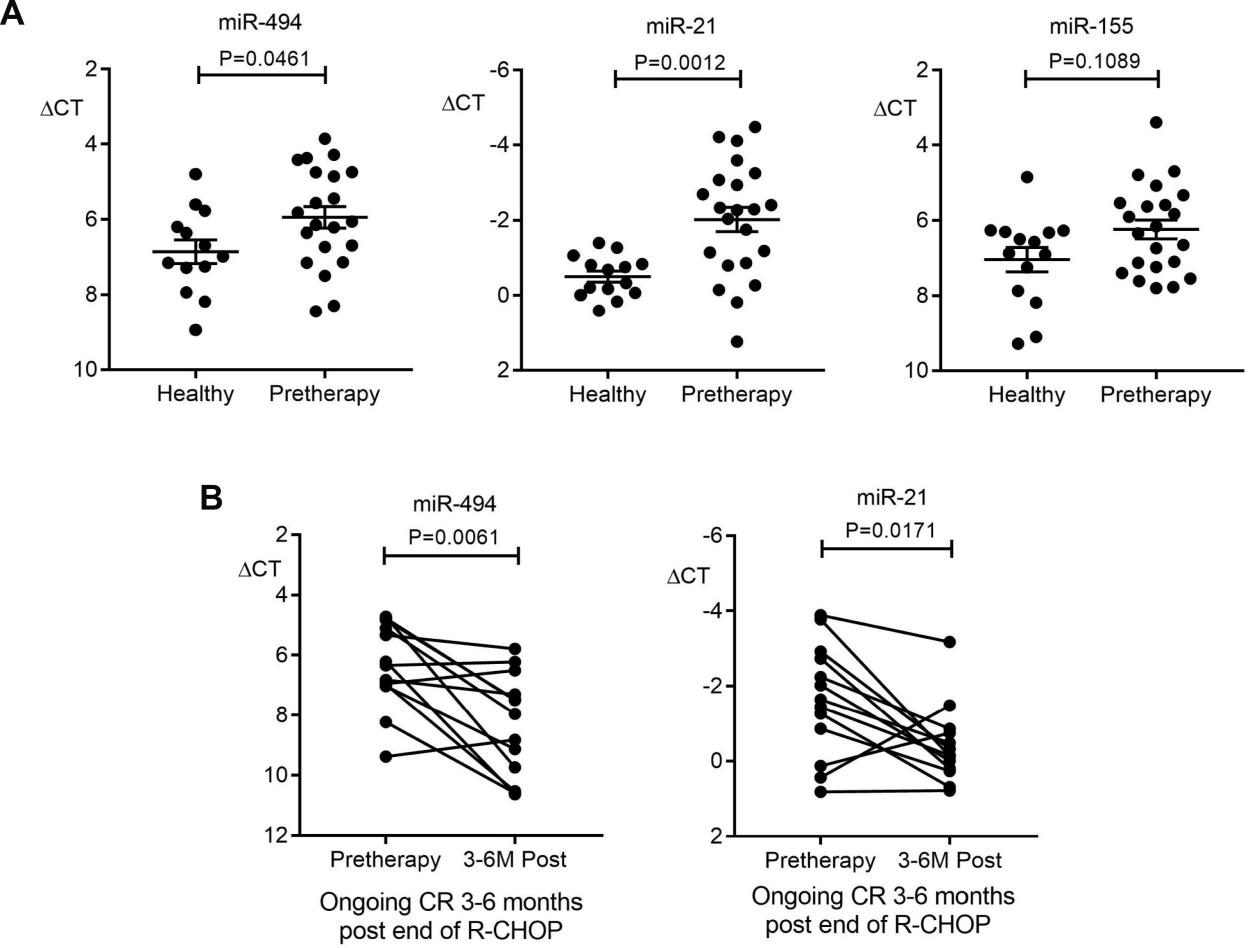
Plasma miR-494 and miR-21 as disease response biomarkers in the discovery cohort (**A**) Comparison of miR-494, miR-21 and miR-155 levels in plasma between pre-therapy (*n* = 21 for miR-494, *n* = 22 for miR-21 and miR-155) and healthy controls (*n* = 13 for miR-494, *n* = 14 for miR-21 and miR-155). Mann–Whitney test was used. (**B**) miR-494 and miR-21 expression in paired pre-therapy versus 3-6 months R-CHOP post-therapy (*n* = 13, Wilcoxon signed rank test). Error bars, mean with SD.

Next, plasma miRNA were quantified by qRT-PCR as disease response biomarkers. In the plasma discovery cohort, there were 22 patients with pre-therapy samples prior to commencing R-CHOP, all of whom entered CR. Thirteen of the 22 had paired plasmas taken pre-therapy and 3–6 months post-therapy for plasma miRNA disease response biomarker assessment. Analysis of pre-therapy samples found pre-therapy miR-494 and miR-21 but not miR-155 were significantly elevated relative to healthy control plasma (new Figure 1A). Therefore, paired testing was restricted to miR-494 and miR-21. This showed that both miRNAs had reduced by 3–6 months post-therapy (Figure 1B) in line with these miRNA having a role as disease response biomarkers.

**Figure 2:**
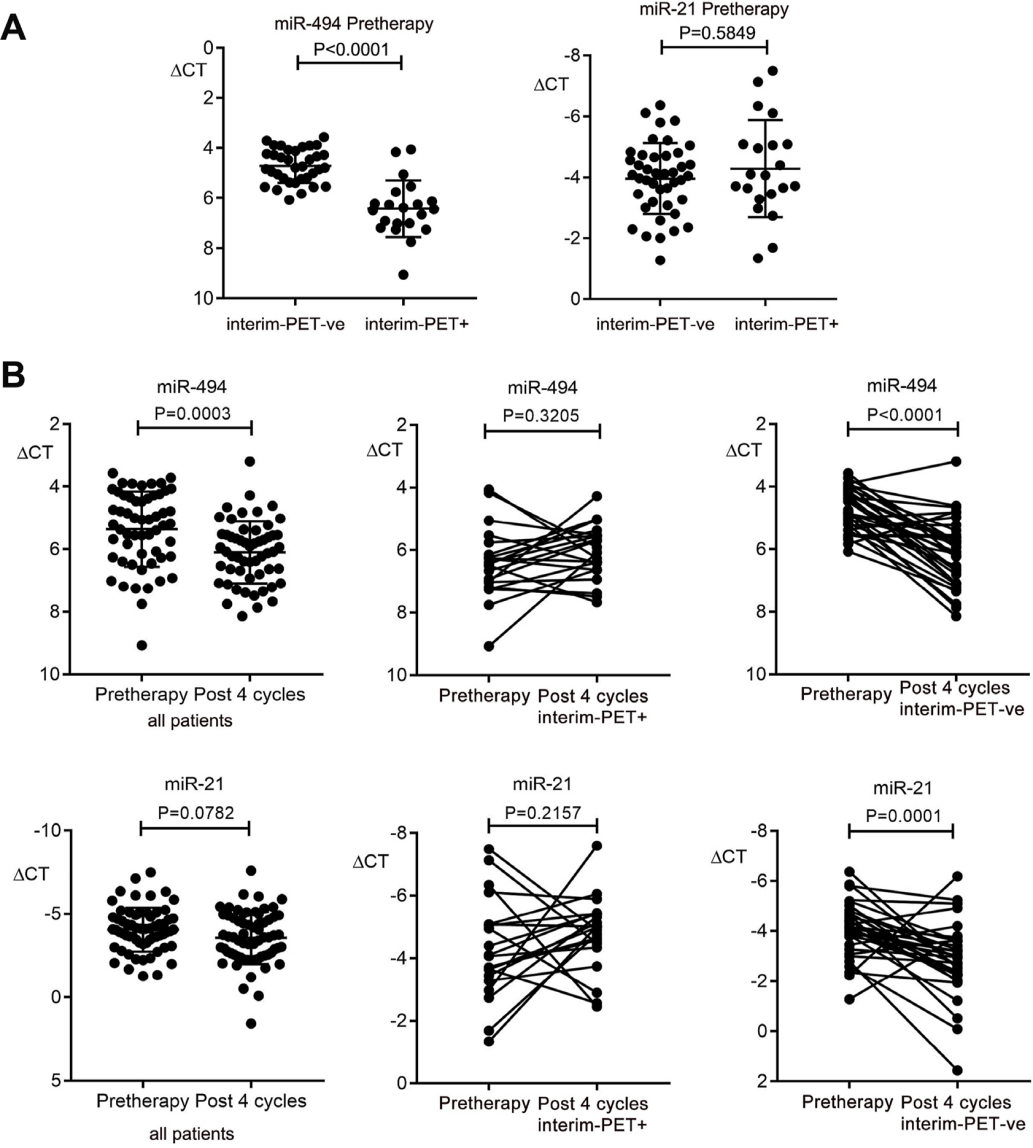
Kinetics of plasma miR-494 and miR-21 expression in DLBCL plasma in the validation cohort (**A**) Comparison of pre-therapy miR-494 and miR-21 between patients that become interim-PET/CT negative (PET-ve) (*n* = 35) versus patients that remain positive (PET+) (*n* = 21). (**B**) Comparison between pre-therapy and post 4 cycles of R-CHOP for all patients (*n* = 56), and in paired samples for interim-PET/CT-ve (*n* = 35) and for interim-PET/CT+ (*n* = 21) for miR-494 and miR-21. Wilcoxon signed rank test was used for paired patient samples, Mann–Whitney test for other comparisons. Error bars, mean with SD.

A plasma validation cohort was used to further investigate the role of plasma miR-494 and miR-21 as biomarkers in DLBCL. Here, qRT-PCR was used to quantify miR-494 and miR-21 in paired plasma samples from 56 patients taken from the ALLG-NHL21 prospective clinical trial, including 35 that achieved interim-PET/CT-ve status (Deauville 1–3) [34], and 21 that did not. First, pre-therapy miRNA were tested as predictive biomarkers of interim-PET/CT status. This showed that pre-therapy levels of plasma miR-494 but not miR-21 were differentially associated with interim-PET/CT status, with miR-494 levels *higher* in patients that were destined to become interim-PET/CT-ve than those that remained interim-PET/CT+ (Deauville 4–5, Figure 2A). This indicates that miR-494 but not miR-21 appears to serve as a c-f circulating biomarker predictive of interim-PET/CT status.

Next, the kinetics of miRNA as disease response biomarkers was tested. In all patients combined, plasma miR-494 but not miR-21 levels reduced between the pre-therapy and cycle 4 R-CHOP time-point (Figure 2B). Importantly, whereas plasma miR-494 levels declined significantly in patients achieving interim-PET/CT-ve status, levels remained *unchanged* in those that remained PET/CT+ (Figure 2B). Similar results were seen with miR-21, indicating that both miRNA appear to serve as disease response biomarkers.

In both discovery and validation plasma cohorts, pre-therapy levels were not associated with age, gender or stage (all *P* = NS). Furthermore, no associations were seen when cohorts were combined (all *P* = NS).

### Immunosuppressive monocytes upregulate miR-494

**Figure 3:**
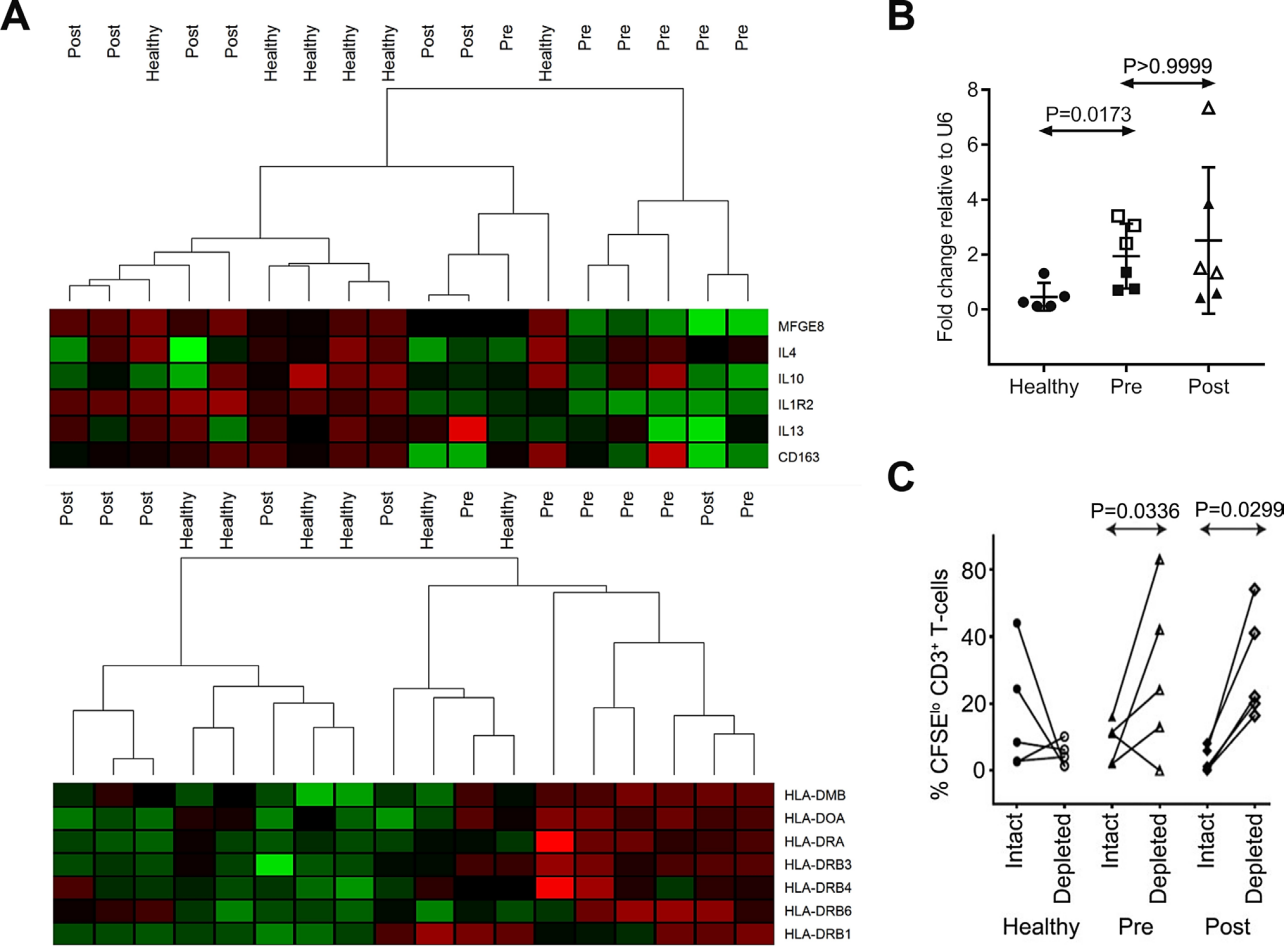
Immunosuppressive features of patient monocytes and miR-494 (**A**) Heat map analysis of gene expression microarray data for circulating monocytes isolated from healthy controls (*n* = 5) and DLBCL patients at pre-therapy (Pre, *n* = 6) and post 4 cycles (Post, *n* = 6). (**B**) miR-494 expression in monocytes used for microarray (1 of 6 health control samples had insufficient RNA for testing, *n* = 5 for healthy, *n* = 6 for patients). Expression is normalized to U6. “□” and “Δ” represent interim-PET/CT+ patients, “■” and “▲” represent interim-PET/CT-ve patients in the right two panels. Mann–Whitney test was used. Error bars, mean with SD. (**C**) CD3+ T-cell proliferation with monocytes depleted (depleted) or not (intact) in healthy (*n* = 4) and DLBCL patient blood samples (*n* = 5). The Mann–Whitney test was used.

Pre-therapy PBMC was available to quantify circulating CD14+HLA-DR^lo^ moMDSC in a subset of patients from the ALLG-NHL21 validation cohort. As expected, this showed that moMDSC were elevated pre-therapy (median 125.6, range 4.73–618.32 per μl) compared to healthy participants (median 72.78, range 0.09–515.84 per μl, *P* = 0.0071 by Mann–Whitney test). As moMDSC were enriched in patient PBMC, we isolated total CD14+ monocytes for further analysis. Gene expression microarray on total CD14+ monocytes isolated from 6 healthy participants and paired pre-therapy/post-cycle 4 monocytes from 6 NHL21 patients, showed distinct clustering of healthy/post-cycle 4 monocytes versus pre-therapy, with up-regulation of antigen presentation and down-regulation of TH2 cytokine and tumor-associated macrophage (TAM) genes in healthy/post-cycle 4 monocytes (Figure 3A). Results were consistent with pre-therapy isolated monocytes having an immunosuppressive profile. Next, miR-494 was quantified by qRT-PCR in the same isolated monocytes that were used for gene expression. This showed that levels of miR-494 were raised in pre-therapy monocytes compared to healthy participants but remained elevated in post-cycle 4 monocytes (Figure 3B). Functional assays showed proliferation of *ex-vivo* stimulated T-cells were higher following depletion of monocytes in patient samples but not in healthy controls (Figure 3C). However, in contrast to the gene expression data but consistent with the persistence of cellular miR-494, functional immunosuppression was observed at both pre-therapy and post-cycle 4 time-points.

We then searched for putative targets of miR-494 in significantly down-regulated genes (*P* < 0.05 with false discovery rate < 0.1) in pre-therapy monocytes relative to healthy monocytes and found 6 genes (*ASXL2, YY1, CAST, RBM23, BRIP1, HSPA13*) that could be potentially regulated by miR-494 (GEO accession number: GSE106997). Interestingly two of these genes are involved in chromatin remodelling (*ASXL2, YY1*) and another in DNA repair (*BRIP1*), implicating miR-494 as potentially having a critical role as a master regulator of transcriptional modification of several pathways underlying moMDSC function.

### MiR-494 is differentially distributed within plasma fractions

**Figure 4:**
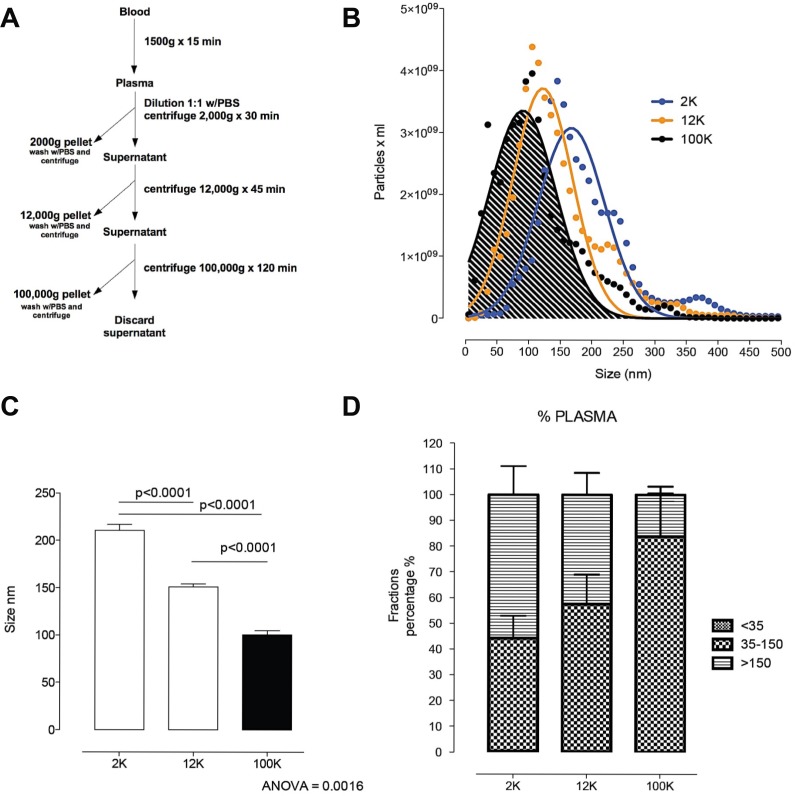
Circulating nanoparticle fractions recovered in successive differential ultracentrifugation pellets (**A**) Scheme of fraction isolation by differential centrifugation from plasma. (**B**) Size distribution of the 2K, 12K and 100K pellet by nanoparticle tracking analysis. (**C**) Comparison of the size of 2K, 12K and 100K pellet. *Post hoc* analysis (multiple-comparison Bonferroni correction test) following one-way ANOVA. (**D**) Size distribution of vesicles <35 nm, 35–150 nm and >150 nm in the 2K, 12K and 100K pellets presented as percentage of the total population of vesicles. Error bars, mean with SD.

Differential centrifugation was used to fractionate plasma from 10 patient and 10 healthy samples (Figure 4A). Heterogeneity in size of c-f nanoparticles recovered by differential centrifugation from these patients was analysed by Nanoparticle Tracking Analysis (NTA). Pelleted materials recovered by low (2,000 × g_av_=2K) or medium (10,000 × g_av_=10K) centrifugation speed were washed and the size distribution was compared with the ultracentrifugation pellet (100,000 × g_av_=100K) (Figure 4B–4D). Interestingly, a majority of nanoparticles pelleting at 10K and at 100K were of a size generally described for exosomes (i.e. 35–150 nm) with ~50% and 85% of the total particles between the exosome size, and about 50% and 20% of the remaining nanoparticles larger than 150 nm. In contrast, the majority of nanoparticles pelleting at 2K were greater than 130nm, which is generally described for micro- and macro-vesicles. Then the concentration of nanoparticles in the pellets was analysed. There was no difference (*P* = NS) in the total number of circulating nanoparticles between DLBCL patients compared to controls.

**Figure 5:**
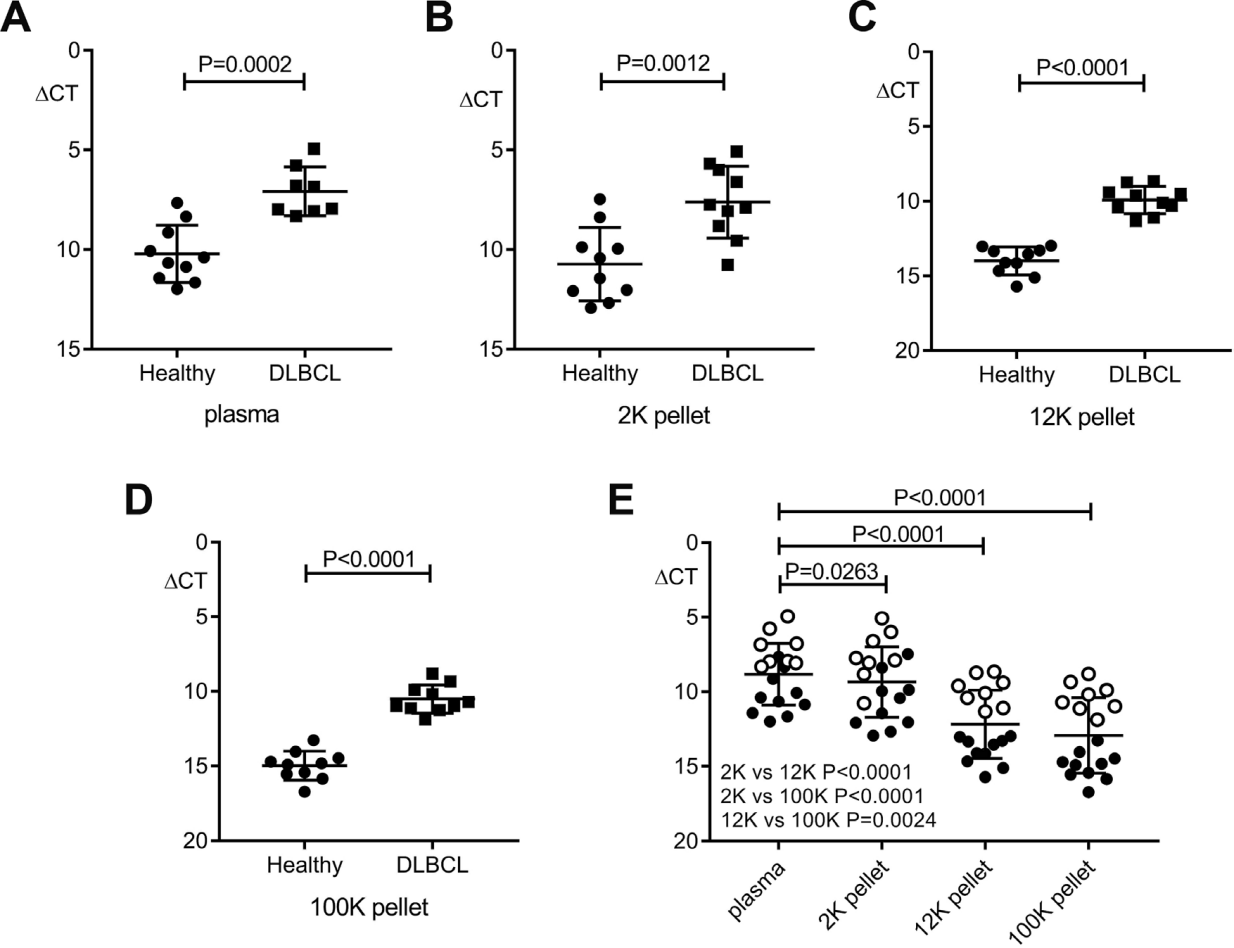
Comparison of miR-494 expression in patient plasma and nanoparticle fractions versus healthy compartments QRT-PCR results are reported by relative delta Ct (ΔCT) normalized to cel-miR-39. (**A**–**D**) Significantly higher miR-494 level in pre-therapy DLBCL (*n* = 8 for plasma, *n* = 10 for each pellet) versus healthy (*n* = 10) samples (unpaired *t* test). (**E**) Differential distribution of miR-494 in plasma and nanoparticle fractions (paired *t* test). *n* = 18 for plasma, *n* = 20 for each pellet. “○” represent patients, “●” represent healthy controls. Error bars, mean with SD.

MiR-494 was then quantified by real-time PCR (qRT-PCR), with results normalized to the spike-in control. This showed that miR-494 was detectable in unfractionated plasma and all sub-fractions, and that in each compartment results were higher in the patient samples (Figure 5A–5D). Levels in plasma were highest, and then reduced progressively from the 2K through to 12K and then 100K pellets (Figure 5E). This indicates that miR-494 is present in all c-f nanoparticle fractions, but is most readily detectable by quantifying unfractionated plasma, confirming that unfractionated plasma is most appropriate for quantification of miR-494.

## DISCUSSION

In this study, we show that c-f miR-494 and miR-21 are elevated in untreated patients with DLBCL, and are disease response biomarkers, with differential reduction in patients that achieve interim-PET/CT negativity after four cycles of R-CHOP compared to those that remain interim-PET/CT positive. MiR-494 is enriched within immunosuppressive monocytes in patients with DLBCL. MiR-494 was present in all plasma nanoparticle fractions but was most readily quantified in unfractionated plasma.

The current gold-standard for cancer detection in blood is circulating tumor DNA (ctDNA) [35–37]. The approach is attractive, as it reflects tumor from all sites and can detect actionable genetic aberrations. One advantage of miRNA as a liquid biomarker (compared to ctDNA) is that it obviates the need for costly and time-consuming personalized gene mutation analysis of mutations present within the malignant B-cell. Against this c-f miRNA - which potentially reflects miRNA from malignant, TME and non-malignant tissue related cells -lack the specificity that circulating tumor DNA can provide [38]. Perhaps the chief role of c-f miRNA is to provide important mechanistic data on the underlying patho-immunobiology of the tumor and the TME and its relationship to circulating immunity, especially as miRNA manipulation is a potential therapeutic avenue.

The TME in DLBCL inhibits the ability of T-cells to kill malignant B-cells [39, 40]. MoMDSC and TAMs are implicated in this suppression [41]. TAMs are enriched within the TME and are believed to facilitate circulating tumor cell seeding of distant metastases in breast, pancreatic and prostate cancers [42]. The relationship between moMDSC and TAMs is incompletely understood, but it is known that following adoptive transfer of moMDSC into tumor-bearing mice, cells with the characteristics of TAMs can be recovered from the TME [43].

MiR-494 has been shown to be upregulated within moMDSC in murine tumor models but human data is lacking [26]. In cHL we have previously shown that c-f miR-494 has utility as a liquid biopsy biomarker that reflects radiographic outcome to induction chemotherapy [16]. Although it has been shown that miR-494 is enriched within tonsillar memory B-cells and in the tissue in follicular lymphoma [21], the current study is the first to focus on miR-494 in DLBCL. Our findings show that in patients with DLBCL, circulating pre-therapy monocytes were phenotypically and functionally immunosuppressive, and importantly were enriched in miR-494. Previously, we have shown that moMDSC decline once R-CHOP therapy has been commenced [23, 25]. Put together, these findings are consistent with moMDSC potentially contributing to the raised plasma miR-494 levels in DLBCL. To further clarify this issue, future studies are needed in which miR-494 is quantified within isolated moMDSC (rather than isolated total CD14+ monocytes) and compared to levels in isolated malignant B-cells as well as in other cellular subsets, and levels of released miRNA from cell types established.

C-f miRNAs are enriched within c-f nanoparticles including extracellular vesicles (EVs) [44]. EVs may serve as delivery vectors to mediate intercellular communication for the selective release and transport of miRNA from a donor cell to neighbouring cells so as to influence their function and enhance tumor cell survival [45–47]. Recent studies highlight the utility of miRNA quantification within specific EV compartments in the diagnosis and therapeutic monitoring of cHL and other B-cell lymphomas [10, 20, 30, 48–50]. There is little data, however, regarding the distribution of miRNA within different c-f nanoparticle fractions in DLBCL, particularly those miRNA that are implicated in the biology of moMDSC, and whether unfractionated plasma or a specific fraction is most appropriate for their quantification. We found that miR-494 was most readily quantified in unfractionated plasma. It was detectable in all nanoparticulate fractions but was enriched within larger nanoparticles relative to smaller particles such as exosomes. Because miRNAs are released extracellularly by selective cellular mechanisms, levels of released miRNA need not necessarily reflect the abundance of miRNA in the cell of origin. Whether nanoparticles enriched in miR-494 are differentially secreted from specific cell-types should be further explored. EVs nanoparticles have been demonstrated to influence host anti-tumoral immunity [51]. Another remaining challenge is to determine whether delivery of miR-494 within specific classes of EVs contributes to the immune modulation mediated by moMDSC to neighbouring cells.

It has been repeatedly recognized that c-f and PBMC miR-21 expression levels are elevated at diagnosis in DLBCL, with conflicting results regarding circulating miR-21's prognostic value [13, 28–31]. To our knowledge, no studies of sequential c-f miR-21 once immuno-chemotherapy has been initiated and comparison with interim-PET/CT have been performed. This is despite emerging data shows that interim-PET/CT is both prognostic and independent of the international prognostic index [52]. We found c-f miR-21 was higher at diagnosis and then reduced in patients that entered CR with ongoing remission 3–6 months after completion of R-CHOP. Next, we showed in a separate cohort taken from a prospective clinical trial, that (as with miR-494) miR-21 declines in response to therapy in responders (as assessed by PET/CT) but remains high in those whose lymphomas remain PET/CT avid. However, unlike miR-494, pre-therapy miR-21 levels were *not* differentially expressed between those destined to become interim-PET/CT-ve or remain interim-PET/CT+. The pre-therapy findings are broadly similar to two prior studies, which showed that miR-21 was not associated with differential chemosensitivity, as evidenced by no difference between those that enter CR versus those with primary refractory disease [32, 53]. Only a subset of patient samples were available for analysis from ALLG-NHL21. Furthermore, because the ALLG-NHL21 was a risk-adapted study, involving dose-intensification in patients with Deauville 4–5 interim-PET/CT and continuation of R-CHOP in patients with Deauville 1–3, the cohort is not designed to test the prognostic impact of pre-therapy miR-21 or miR-494 on progression free or overall survival. To test this association, prospective studies in uniformly treated patients are required. Studies testing the differential expression of circulating miR-21 between cell-of-origin (COO) DLBCL sub-types have been conflicting. One study found serum miR-21 elevated in the activated B-cell subtype [30], however this was not replicated by other investigators [53]. Unfortunately COO was only available in a minority of patients in the ALLG-NHL21 validation therefore an analysis for correlation between COO and miR-21 could not be performed.

In conclusion, miR-494 and miR-21 are disease response biomarkers in DLBCL. MiR-494 is a moMDSC associated miRNA that appears to be upregulated in the diagnostic tissues and cell-free circulation of patients with DLBCL, where it shows utility as a biomarker of moMDSC kinetics and immunosuppressive activity. Findings require independent validation and the prognostic impact of c-f miR-494 should be prospectively tested. The role of miRNA in the regulation of the molecular networks that govern moMDSC, and the exploration of miRNA manipulation as a potential therapeutic requires further elucidation in mechanistic studies.

## MATERIALS AND METHODS

### Patient characteristics

For miRNA quantification on diagnostic biopsies, 99 DLBCL tissue samples were chosen, with the sole criteria being formalin-fixed, paraffin-embedded (FFPE) tissue availability for RNA extraction. All patients were from the Princess Alexandra Hospital (Queensland) or the Canberra Hospital (Australian Central Territory) as previously published [6]. Only *de novo* cases of DLBCL were included. Grade IIIB or transformed follicular lymphoma, HIV-positive and post-transplant patients were excluded. Twenty non-diseased normal lymph nodes obtained from individuals without lymphoma were also analysed (ProteoGenex Inc.). The discovery cohort that was used to test c-f miRNA in sequential samples were 22 DLBCL patients (median age 61 years, range 23–82; stage I-II 38%), enrolled as part of a prospective observational study in which patients had plasma taken at diagnosis (pre-therapy), with 13 patients also having plasma taken at 3–6 months following cessation of ‘R-CHOP’ (rituximab, cyclophosphamide, doxorubicin, vincristine, prednisolone) immuno-chemotherapy [37]. For validation we tested an independent cohort of 56 patients (median age 55 years, range 23–70; M:F 36:20; stage I-II 24%) in which paired plasma samples were available from patients with DLBCL enrolled in the Australasian Leukaemia Lymphoma Group NHL21 (ALLG-NHL21) clinical trial (ACTRN-12609001077257) [54]. In that study, blood was taken prior to cycle 1 of R-CHOP and following the 4th cycle of R-CHOP at time of interim-PET/CT restaging performed on day 17–20 of cycle 4 (after which treatment was risk-adapted by interim-PET/CT status). The PET/CT was scored centrally (Deauville score). Further therapy was dependent on the outcome of the interim-PET/CT scan. Patients with Deauville 1–3 continued with 2 further cycles of R-CHOP and then 2 further cycles of rituximab. Patients with Deauville 4–5 received R-ICE (rituximab, ifosfamide, carboplatin, and etoposide) chemotherapy for 3 cycles followed by 90Y-ibritumomab tiuxetan–BEAM (BCNU, etoposide, cytarabine, and melphalan) and autologous stem cell rescue. The study conformed to the Declaration of Helsinki and was approved by the University of Queensland Human Research Ethics Committee and written informed patient consent was obtained at participating sites.

### miRNA lymph node and RNA monocyte arrays

Tissue RNA was extracted from FFPE tumor biopsies using RecoverAll Total Nucleic Acid Isolation kit (Ambion). NanoString nCounter (NanoString Technologies, Seattle, USA) was conducted as previously described [6, 23, 55], using nCounter Human v.2 miRNA Expression panel for analysis of 800 miRNAs derived from miRBase v.18. Total RNA was used as input for nCounter miRNA sample preparation reactions. All sample preparation and processing were performed according to the manufacturer's protocol. Following ligation, sample preparation reactions were purified and diluted, and hybridization reactions were performed by incubation at 65°C for 20 hours. The target and probe complexes were washed and immobilized in the cartridge, and the amount of miRNA quantified on the nCounter Prep Station and Digital Analyzer nCounter.

The Agilent miRNA microarray used 100 ng of total RNA, and utilized “Human microRNA-Microarray Release 16.0, 8 × 60K” (Ramaciotti Centre, Sydney, Australia) as previously described [56]. This array tests for 1262 human microRNA. Normalization to background was performed by Agilent's “feature extraction software” followed by a quantile normalization using “GeneSpring GX software”.

For monocyte gene expression array, RNA was extracted from FACS-sorted CD14+ monocytes and analysed using Illumina Human HT12v4 Bead Array for whole genome expression. The whole genome expression data is available from GEO (GSE106997). The data was pre-processed and analysed in R environment using the Bioconductor limma package [57]. Expression values were quantile normalised and the linear model fitting was applied for DE analysis. Clustering based on gene expression values were generated in R environment.

### Isolation of c-f nanoparticles by differential centrifugation

Nanoparticles were isolated from 500 μL of plasma. Sub-populations of c-f nanoparticle subtypes were enriched by differential centrifugation steps as previously described [58]. Plasma was diluted with an equal volume of phosphate-buffered saline (PBS) at pH 7.4, and centrifuged at 2,000 × g_av_ for 30 minutes at 4°C (nuclear fraction; Heraeus Fresco 21, Thermo Fisher Scientific). Subsequently, the 2,000 × g_av_ supernatant was centrifuged at 12,000 × g_av_ for 45 minutes at 4°C (mitochondrial fraction). The supernatant was centrifuged at 100,000 × g_av_ for 2 hours (microsomal fraction; WX Ultra 100, Thermo Fisher Scientific). All pellets were washed in 50–60 mL of PBS and re-centrifuged at the same speed before being resuspended in 50–100 μL of sterile PBS and stored at −80°C.

### Nanoparticle tracking analysis (NTA)

NTA measurements were performed using a NanoSight NS500 instrument (NanoSight, UK) as previously described [59]. 2K, 12K and 100K pellets were diluted to 100 μg/ml and videos processed and analyzed. A minimum of 200 completed tracks per video were collected for each analyzed sample. NTA post-acquisition settings were optimized and kept constant between samples. Each video was analyzed to give particle size together with estimated number of particles per mL plasma or per 10^6^ cells. 100 nm polystyrene latex microspheres (Malvern) were routinely analyzed to confirm instrument performance.

### Plasma miRNA extraction and quantitative real-time PCR

Total RNA was extracted using miRNeasy Serum/Plasma Kit (Qiagen). Cel-miR-39-3p, cel-miR-248 and osa-miR-414 were added as spike-in miRNA oligo after lysis during RNA extraction. Using the Qiagen miScript PCR system, including miScript II RT Kit and SYBR Green PCR Kit, cDNA was reverse transcribed from 5μL of RNA and then diluted with 10-fold volume of H_2_O. Plasma miRNAs were quantified on ViiA™ 7 Real-Time PCR System (Thermo Fisher Scientific). Each reaction contained 1 μL of diluted cDNA, all run in duplicate or triplicate 10 μL reactions. Equal volumes were used throughout to reduce technical variation. Comparative quantification was used to determine relative expression of miRNA, shown as delta Ct after normalization. To quantify miR-494 level in nanoparticle fractions and input plasma, delta Ct was corrected to account for the volumetric differences to allow for results to be directly comparable.

### Immune assays

To test the immunosuppressive effects on T-cells by circulating monocytes *ex-vivo*, PBMC (either monocyte replete or depleted using immuno-magnetic selection [EasySep, StemCell Technologies]), were carboxyfluorescein-succinimidyl-ester (CFSE) labelled and stimulated with anti-CD3/CD28/CD137 beads (Invitrogen, California, USA) at a bead: cell ratio of 1:10. Cells were cultured for 96 hours and T-cell proliferation assessed by flow cytometry. Data was acquired using a BD LSRII flow cytometer with FACSDiva software (BD, Australia). Data analysis was performed on compensated data using FlowJo 10.x software (Tree Star, USA).

### Statistical analysis

Data from the Agilent microRNA array and Nanostring nCounter assay were analyzed using Agilent GeneSpring GX (v.13.1.1) and nSolver™ software (3.0), respectively. Array data was normalized to the data-point of the 75th percentile. NanoString expression counts were normalized to the geometric mean of the top 100 miRNAs in all samples. The differences between the means of experimental groups were analysed by a two-tailed Mann–Whitney rank sum test (with Benjamini-Hochberg false discovery rate testing). Only statistically significant miRNAs (*P* ≤ 0.05) with fold change ≥5 across both methods were selected for further analysis. Scatter plots were generated using GraphPad Prism 7.0 (GraphPad Software, CA, USA). The target prediction for miR-494 was performed using TargetScan (release 7.1) to identify down-regulated genes (*P* < 0.05 with false discovery rate < 0.1) in pre-therapy monocyte compared to healthy participants [60].

### Data availability

All data generated or analyzed during this study are included in this published article and the GEO database (accession number: GSE106997).
